# A sting in the tail—are antibodies against the C‐terminus of *Plasmodium falciparum* circumsporozoite protein protective?

**DOI:** 10.15252/emmm.202317556

**Published:** 2023-04-21

**Authors:** Jem Murdoch, Jake Baum

**Affiliations:** ^1^ School of Biomedical Sciences UNSW Sydney Kensington NSW Australia

## Abstract

Malaria remains a huge burden on global public health. Annually there are more than 200 million cases with > 600,000 deaths worldwide, the vast majority of which occur within Sub‐Saharan Africa (WHO; World Malaria Report, 2021). Malaria disease is the consequence of infection by a protozoan parasite from the genus *Plasmodium* with most morbidity and mortality caused by *P. falciparum.* With rates of infection plateauing and rebounding in some areas (in particular, as a result of the disruption caused by the COVID‐19 pandemic), there have been increasing calls for new initiatives that can reduce malaria incidence towards local elimination or the hoped for goal of global eradication. In 2021, the World Health Organisation approved the first malaria vaccine RTS,S/AS01 (also called Mosquirix™), indicating it to be safe for use in young children and advocating its integration into routine immunisation programmes. Approval of this vaccine clearly represents a major landmark in global efforts towards malaria control and eradication aspirations. RTS,S modest efficacy, however, points at the need to better understand immune responses to the parasite if we hope to design next generation malaria vaccines with increased potency.

## Graphical abstract

J. Baum and J. Murdoch discuss the study by Opeyemi Oludada *et al* (in this issue of EMBO Mol Med) that investigates the molecular characteristics and protective potential of human monoclonal antibodies against the highly immunogenic *Plasmodium falciparum* circumsporozoite protein (PfCSP) C‐terminal domain.

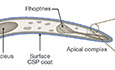

Work starting in the 1970s demonstrated protection from malaria challenge following immunisation with radiation‐attenuated sporozoites, the parasite form delivered during an infectious mosquito bite (Clyde, 1990). Subsequent work validated that a key mediator of protection was the sporozoite surface circumsporozoite protein (CSP), a glycophosphatidylinositol anchored protein, present at ~1 million copies on each sporozoite (Fig 1). As the dominant immunogen of the sporozoite, and with a significant proportion of protective immunogenicity located in the repeat regions of the CSP protein (Zavala *et al*, 1985), the idea of a subunit vaccine took shape aiming to mimic protection afforded by whole parasite vaccination. Nearly 40 years in the making, this gave rise to RTS,S/AS01, developed initially out of a collaboration between GlaxoSmithKline (GSK) and the Walter Reed Army Institute of Research (WRAIR). RTS,S is a formulation comprising a truncated CSP fused together with hepatitis B surface antigen (HBsAg). Co‐expression with excess HBsAg forms virus‐like particles (VLPs), which through various iterations was eventually co‐formulated with an adjuvant AS01. The truncated CSP construct contains an 18.5 asparagine–alanine–asparagine–proline (NANP) repeat (Zavala *et al*, 1985), acting as an immunodominant B cell epitope (Fig 1). CD4^+^ and CD8^+^ T cell epitopes are also present within the C‐terminus of CSP. The NANP repeat induces a strong IgG antibody response which is thought to be the main mediator of protection (Beeson *et al*, 2022).

**Figure 1:**
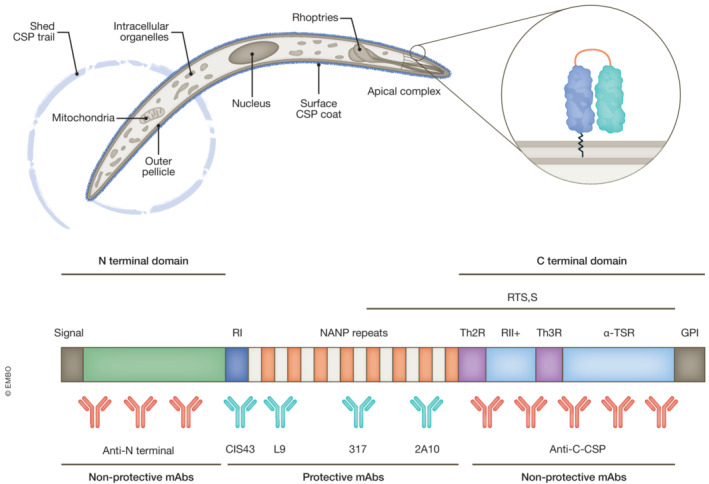
The circumsporozoite protein, CSP, coats the outer surface of *Plasmodium* sporozoites with ~ 1 million copies per cell The C‐terminal TSR domain is inaccessible to antibodies, shielded by the N‐terminal domain and likely only revealed by processing *8*. Proteolytic cleavage of the N‐terminal domain at RI (the junctional region) just before parasite entry into hepatocytes may indicate an immune evasion strategy by the parasite to shield its key cell‐surface‐binding/interacting domain, minimising its exposure to immune surveillance. Anti‐C‐terminal (from Oludada *et al*, 2023) and anti‐N‐terminal antibodies (previous work) are for the most part non‐protective, in contrast to the well‐characterised CIS43, L9, 317 and 2A10 antibodies which confer protection via their targeting of the exposed repeat domains.

RTS,S/AS01 protection is, however, only relatively modest. Initial phase 3 clinical trial across seven sub‐Saharan African countries demonstrated efficacy against clinical malaria of ~ 20% in infants aged 6–12 weeks and ~ 35% in children aged 5–17 months, although this dropped significantly after 18 months necessitating a booster (RTS,S Clinical Trials Partnership, 2015). Given the scale of the burden of malaria disease globally (WHO, 2021), RTS,S is still predicted to have a massive impact on global health (Penny *et al*, 2016). However, its modest efficacy clearly demands development of additional effective, long‐lasting malaria vaccines^4^.

## C‐terminus antibodies

One core goal of current efforts towards future malaria vaccine development is to better understand the nature of protection afforded by RTS,S/AS01 immunisation (and general strategies aimed at targeting the sporozoite). A significant portion of recent efforts have focussed on investigating the protective efficacy of human monoclonals (mAbs) against PfCSP that arise post‐vaccination. Oludada *et al* (2023), in this issue of EMBO Molecular Medicine, have returned to basics to explore the molecular characteristics and protective potential of mAbs against PfCSP following whole sporozoite immunisation. Their unique take, however, is the exclusive focus on the C‐terminal domain of the protein, CSP's “tail”. Protective mAbs towards the N‐terminal junction and repeat regions have been well characterised, however, the role of mAbs towards the terminal N and C domains of CSP is less well understood. As well as being poorly immunogenic, the PfCSP N‐terminus undergoes proteolytic cleavage during parasite infection (Coppi *et al*, 2005), with the few known specific mAbs characterised showing little to no sporozoite reactivity or parasite inhibitory activity. In contrast, the C‐terminal domain induces a dominant humoral response upon recombinant PfCSP immunisation. However, the limited number of human C‐terminal‐reactive mAbs described to date has prevented a more in‐depth analysis of their gene features, epitope breadth and parasite inhibitory activity. To address this gap in knowledge, Oludada *et al* sourced serum samples from 12 malaria‐naive European volunteers immunised with 900,000 cryopreserved radiation‐attenuated *P. falciparum* sporozoites (the Sanaria^®^ PfSPZ vaccine based on the NF54 parasite strain; Mordmüller *et al*, 2022).

To compare the anti‐C‐CSP and anti‐NANP antibody responses, Oludada *et al* isolated the circulating PfCSP‐reactive memory B cells (towards immunoglobulin characterisation) from all donors 14 and 35 days after their third immunisation. Immunoglobulin variable heavy‐chain genes (IGVH) undergo somatic hypermutation (mutation rate 10^6^‐fold higher than background rate), which means each B cell population ends up with a distinct heavy‐chain variable region. Oludada *et al* discovered that genes encoding variable heavy chains VH3‐21 and VH3‐33 were highly enriched in PfCSP‐reactive memory B cells. VH3‐33‐containing antibodies were associated with an IgM response. Mature yet naive B cells express IgM, which have a lower affinity for antigens, but due to the pentameric structure have a higher avidity. In contrast, the VH3‐21 genes were associated with IgG response. IgG is a bivalent immunoglobulin with four subclasses (IG1‐4). It is the product of an IgM B cell undergoing isotype‐switching recombination and affinity maturation (characterised by a higher somatic hypermutation rate) within germinal centres. IgG antibodies typically have substantially higher affinities for antigens than IgM antibodies.

Through their screen, Oludada *et al* produced 73 C‐CSP mAbs and 102 NANP mAbs respectively. One C‐CSP, mAb showed signs of cross‐reactivity towards both NANP and the N‐junction region, whereas the rest of the anti‐C‐CSP mAbs were highly specific, with half the anti‐C‐CSP mAbs encoded by VH3‐21. In contrast, a significant proportion of anti‐NANP mAbs showed cross‐reactivity with the N‐junction and were mostly encoded by VH3‐33 genes. These NANP repeats may mediate cross‐linking of multiple B cell receptors, which would bias the response towards an IgM VH3‐33 response. Alternatively, the structural differences between the disordered yet flexible NANP repeat region and the structured C‐terminal domain may explain the epitope‐associated differences in affinity maturation. Investigating the binding characteristics further, only three C‐CSP mAbs showed reactivity towards linear epitopes, while the vast majority shared specificity for identical or overlapping conformational epitopes in the polymorphic Th2R/Th3R regions of CSP, a C‐terminal domain containing a thrombospondin type 1 repeat (TSR) domain (Fig 1). The difference in the abundance between linear and conformational epitope‐binding mAbs may be linked to differences in the frequency of naive precursor B cells or differences in epitope presentation and immunogenicity.

## C‐terminus antibodies lack parasite inhibitory activity

To determine if these anti‐C‐CSP mAbs could inhibit sporozoite activity, Oludada *et al* measured 15 representative antibodies with high C‐CSP affinities and measured their ability to bind directly to the surface of live fluorescent (mCherry) *P. berghei* sporozoites (a rodent malaria model) expressing PfCSP. They found that none of the TSR C‐CSP antibodies bound to live sporozoites, which corroborates previous observations that the TSR domain is not accessible on the surface of sporozoites. This inaccessibility fits with the idea that CSP processing may regulate the exposure of surface epitopes (Coppi *et al*, 2005). Furthermore, the only anti‐C‐CSP antibody that bound strongly was the singular cross‐reactive mAb. As with the other anti‐C‐CSP mAbs, however, it could not inhibit sporozoite activity *in vivo*.

Oludada *et al* compared their results to other well‐established inhibitory anti‐CSP antibodies; mAbs 2A10 and 317 which both target NANP, with the anti‐C‐CSP mAbs showing very limited *in vivo* inhibitory activity even at 100‐fold higher concentrations than 2A10. Furthermore, when compared to the next‐generation inhibitory antibodies CIS43 (N‐terminal junction) and L9 (minor repeat NVDP) mAbs, these data highlight that while the C‐terminus is clearly immunogenic, it appears to be irrelevant in the context of an effective humoral response.

To explore the nature of mAb action, Oludada *et al* also did a deep structural dive into how antibody binding is mediated. Their focus centred on investigating the C‐linker region‐specific mAb 3764, the only C‐CSP mAb encoded by a VH3‐33 gene. Solving the crystal structure of mAb 3764 in complex with its target motif DPN, they demonstrate how the C‐linker domain is recognised by a non‐cross‐reactive human anti‐PfCSP mAb. This shows the steric constraints that inhibit cross‐reactivity, highlighting the importance of peripheral residues within epitopes in mAb binding specificity and cross‐reactivity.

## Outlook

The herculean effort by Oludada *et al* in characterising human mAbs following whole sporozoite immunisation and their characterisation with respect to the C‐terminus could prove fundamentally important in guiding future malaria vaccine design. While there might be a temptation to read this as a negative result (i.e. that C‐terminus antibodies lack *in vivo* parasite inhibitory activity), the work provides some clear insights that may be critical for next‐generation development of RTS,S‐like sub‐unit vaccines based on PfCSP. Of keen interest, their work demonstrates the inaccessibility of the C‐terminus in the native context (on the sporozoite surface) to neutralising antibodies. This provides tantalising insight into the nature of PfCSP on the sporozoite surface, something that has never been visualised (at the structural level) *in situ*. It also raises questions about the inclusion of the C‐terminus in the current RTS,S vaccine design. Should future efforts drop this domain entirely? One caution is the key role that CD4^+^ and CD8^+^ T responses play in protective responses to malaria, and with known T‐cell epitopes within the C‐terminus (Good *et al*, 1988), it may be premature to entirely dismiss CSP's tail in providing effective protection. Their work does throw into question some previous studies on C‐terminal protection, where antibodies targeting the same epitopes as Oludada *et al* were found to provide modest protection (Beutler *et al*, 2022). That these mAbs required a 100‐fold higher dose for protection compared to anti‐NANP mAbs like 2A10 may reconcile some of this discrepancy.

Perhaps the broader question the study raises is whether CSP is still the only (or even best) target for a subunit‐based malaria vaccine, given it is clearly evolved to mitigate an inevitable human immune response. Recent findings have shown that targeting the repeat regions in *Plasmodium* proteins, such as NANP, can lead to less durable antibody responses (Raghavan *et al*, 2023). Coupled with evidence that avid binding of B cells to NANP might even suppress immune responses to protective subdominant epitopes within CSP hints at what has been called the “decoy hypothesis” (Chatterjee *et al*, 2021). That is, CSP NANP repeats act as a smoke screen preventing the immune system from raising a strong effective neutralising response. CSP is shed copiously by the parasite as it migrates. It is highly repetitive and while immunogenic, is not always protective. Could it be that a parasite that has been co‐evolving with humans (indeed across all vertebrate lineages) for millennia has developed the ultimate immunological decoy? Combining the evidence presented here with the modest efficacy of RTS,S/AS01 (RTS,S Clinical Trials Partnership, 2015) perhaps more than anything highlights the need for efforts pursuing novel non‐CSP surface antigens if we are to ever truly overcome this ancient foe.
